# Influence of Laser Powder Bed Fusion Process Parameters on Voids, Cracks, and Microhardness of Nickel-Based Superalloy Alloy 247LC

**DOI:** 10.3390/ma13173770

**Published:** 2020-08-26

**Authors:** Olutayo Adegoke, Joel Andersson, Håkan Brodin, Robert Pederson

**Affiliations:** 1 Department of Engineering Science, University West, SE-461 86 Trollhättan, Sweden; joel.andersson@hv.se (J.A.); robert.pederson@hv.se (R.P.); 2 Materials Technology Additive Manufacturing Product Development-Industrial Gas Turbines, Siemens Industrial Turbomachinery, SE-612 83 Finspång, Sweden; hakan.brodin@siemens.com

**Keywords:** laser powder bed fusion, superalloy, Alloy 247LC, cracks, voids

## Abstract

The manufacturing of parts from nickel-based superalloy Alloy 247LC by laser powder bed fusion (L-PBF) is challenging, primarily owing to the alloy’s susceptibility to cracks. Apart from the cracks, voids created during the L-PBF process should also be minimized to produce dense parts. In this study, samples of Alloy 247LC were manufactured by L-PBF, several of which could be produced with voids and crack density close to zero. A statistical design of experiments was used to evaluate the influence of the process parameters, namely laser power, scanning speed, and hatch distance (inherent to the volumetric energy density) on void formation, crack density, and microhardness of the samples. The window of process parameters, in which minimum voids and/or cracks were present, was predicted. It was shown that the void content increased steeply at a volumetric energy density threshold below 81 $J/{\mathrm{mm}}^{3}$. The crack density, on the other hand, increased steeply at a volumetric energy density threshold above 163 $J/{\mathrm{mm}}^{3}$. The microhardness displayed a relatively low value in three samples which displayed the lowest volumetric energy density and highest void content. It was also observed that two samples, which displayed the highest volumetric energy density and crack density, demonstrated a relatively high microhardness; which could be a vital evidence in future investigations to determine the fundamental mechanism of cracking. The laser power was concluded to be the strongest and statistically most significant process parameter that influenced void formation and microhardness. The interaction of laser power and hatch distance was the strongest and most significant factor that influenced the crack density.

## 1. Introduction

Laser powder bed fusion (L-PBF) is an additive manufacturing (AM) process, in which a laser is utilized to melt a powder on a powder bed or substrate one layer at a time. A powder bed layer can be as thin as 20 µm. The process is conducted in an inert gas atmosphere and the geometry of the part is fed from a 3D computer-aided design (CAD) file. L-PBF gained attention in the manufacturing of gas turbine blades, for which Alloy 247LC (LC stands for low carbon) is a suitable material. This is a precipitation strengthened nickel-based superalloy that has good creep strength, high temperature oxidation, and good corrosion properties [1]. These blades have internal cooling channels with complex designs which are difficult to manufacture using conventional processes; however, they could be manufactured with the L-PBF process. Nevertheless, processing of Alloy 247LC is challenging, as this alloy is susceptible to weld cracking, which is often attributed to the high volume fraction of γ’ precipitates [2,3,4]. High thermal gradients in the range of, e.g., 5–20 K/µm exist in L-PBF [5]. The high propensity of the alloy to cracking is related to the combination of high stresses introduced by repeated steep thermal gradients employed in the L-PBF process, as well as the general cracking susceptibility of the alloy material itself. Hence, to successfully utilize L-PBF to manufacture Alloy 247LC components, the cracking problem should be addressed. The cracking mechanisms can be classified into solidification cracking, liquation cracking, strain age cracking, and ductility dip cracking. Cracks can be strongly influenced by the metallurgical properties of the material; for example, the solidification cracking is more probable when the solidification temperature range is high [6]. More details about the aforementioned cracking mechanisms can be found in [6,7,8]. In this field, the L-PBF processability is synonymous to weldability. The welding literature defines weldability as “the ability to produce parts that are free of defects, such as porosity, lack of fusion, and cracks” [6]. The porosity generation is different from the crack formation mechanism mentioned above. Porosity can originate from entrapped gases in the powder during the powder atomization process, or during the melting process in the LPBF equipment. A lack of fusion primarily occurs, when there is inadequate wetting between a newly formed layer and the preceding layer or substrate to promote epitaxial growth of grains [9]. These defects can deteriorate the mechanical properties of the parts produced. Weldability is further defined as the ability of the processed part to acquire the desired properties and fulfil its service conditions [6]. This requires that the microstructure be preserved during the processing to guarantee favorable properties over a long period of time. The L-PBF process parameters influence the thermal history, which in turn, is location dependent [10]. Thus, the microstructure may vary locally. This is typically observed in AM processes, such as L-PBF, electron beam powder bed fusion (EPBF), and direct energy deposition (DED). Thus, the L-PBF process parameters, including the effect of location, should be fully understood to produce defect-free parts with acceptable microstructure and properties. In this study, the process parameters investigated are the laser power, scanning speed, and hatch distance (henceforth, referred to as power, speed, and hatch, respectively). These were selected primarily because it has been established in the literature that they strongly influence the defect formation and microstructure of components made by L-PBF [2,10,11,12]. These parameters, together with the constant layer thickness, are included in the calculation of the volumetric energy density (henceforth, referred to as energy density) which is given as Equation (1):

(1) Energy density=Pv·h·t

where *P*, *v*, *h*, and *t* are the power, speed, hatch, and layer thickness, respectively. A review of past literature reveals that the investigation by Carter et al. [2,13] was the foremost work that investigated the influence of the L-PBF process parameters on Alloy 247LC. Cuboidal samples of dimensions 10 mm × 10 mm × 20 mm were produced in a Concept Laser M2 system. The process parameters utilized were in the range of 150–200 J/s, 400–2000 mm/s, and 0.2–0.53 for power, speed, and hatch (dimensionless in the Concept laser M2 equipment), respectively. They presented their optimized process parameters to be 150 J/s, 1500 mm/s and 0.3. It was concluded that cracks could not be eliminated completely in the investigated process parameter window. Unlike the work by Carter et al., the current study has used the statistical design of experiment (DOE) methodology. The advantage of this method is that the prediction of a large process window is possible with a relatively few experiments [14]. The DOE is a proven methodology and has been used to optimize various L-PBF processes [11,15,16,17]. This method also presents an opportunity to readily study the interaction of parameters; for example, the interaction of power and speed means that the relationship between the response (void, crack density, or microhardness) and speed could be different, depending on the power level [18]. Therefore, a DOE can provide more insight into the relationship of the responses to the parameters than what is presently available in the literature on L-PBF of Alloy 247LC.

The objective of this work was, therefore, to use the DOE to study the influence of power, speed, and hatch on the number of voids (porosity and lack of fusion), and cracks. These process parameters were investigated to disclose the process windows with the lowest number of defects. In addition, the microhardness of the material was investigated to reveal the correlations with the process parameters and gain knowledge on γ’ precipitation in the microstructure. This is a reasonable approach because the microhardness mainly depends on the precipitation of γ’ in L-PBF-manufactured Alloy 247LC components [19,20,21]. Thus, the results from this study are expected to serve as a guide to minimize the defects when new samples are developed via L-PBF. The knowledge gained from this study can also aid future investigations of the cracking mechanisms.

## 2. Experiment

### 2.1. Statistical Design of Experiment (DOE)

Modde software (MODDE 12, Umetrics, Umeå, Sweden) was used for defining the DOE. The DOE was a $3^{3}$ full factorial design, which means that the three factors, i.e., power, speed, and hatch were varied at three levels for each factor. Thus, the DOE yielded a total of 27 different combinations of the process parameters. These different combinations are called experiments. Three replicates, namely Samples 28, 29 and 30, which have the same parameters as the mid-point sample, i.e., Sample 14, were added, resulting in a total of 30 experiments. The power levels were set to 170, 195, and 220 J/s. The speed levels were set to 2800, 3000, and 3200 mm/s, and the hatch levels were set to 20, 30, and 40 µm. These factor levels were combined in the full factorial design displayed in the first four columns of Table 1. Overall, there were four samples (Samples 14, 28, 29, and 30) which had the same combination of process parameters. The presented parameter combinations were subsequently used to manufacture cube samples described in Section 2.2. After the cubes were manufactured, the responses void, crack density, and microhardness were measured as described in Section 2.3 and Section 2.4. The results were input in Modde and these are displayed in the last three columns of Table 1. The column for energy density is also added in the table, even though it was not used in Modde for the analysis. It is added to Table 1 to indicate which energy density, each of the samples was manufactured. The energy density included the layer thickness of 20 µm as an inherent parameter. A quadratic regression was used to model each response according to the Equation (2) below [14,18].

(2) $$y={\;\beta }_{0}+{\;\beta }_{1}x_{1}+{\;\beta }_{2}x_{2}+{\;\beta }_{3}x_{3}+{\;\beta }_{11}x_{1}^{2}+{\;\beta }_{22}x_{2}^{2}+{\;\beta }_{33}x_{3}^{2}+\beta_{12}x_{1}x_{2}+\beta_{13}x_{1}x_{3}+\beta_{23}x_{2}x_{3}+\epsilon$$

where y is the response—void, crack density, or microhardness, and is the dependent variable; the β terms are constants, of which all except $\beta_{0}$ are called regression coefficients; the x terms are the independent variables denoting the factor levels; for example, $x_{1},{\;x}_{2,\;}{\;x}_{3}$ represent the levels of power, speed, and hatch, respectively, of a particular sample. Furthermore, the combination of $x_{1}x_{2}$ represents the interaction of power and speed. The other combinations also have a similar interpretation. The factor levels represented by x were defined on a coded scale. The power levels of 170, 195 and 220 J/s were coded −1, 0 and 1, respectively. The speed levels 2800, 3000 and 3200 mm/s were coded −1, 0 and 1, respectively. Finally, the hatch levels of 20, 30 and 40 µm were coded −1, 0 and 1, respectively. ϵ is the modelling residual or error. The regression coefficients were calculated in Modde by the method of least squares and a 95% confidence interval of these coefficients was also estimated. To test the regression model, the first analysis carried out in Modde was to compare the variability of the replicates to the variability in all the experiments. This repeatability test was conducted and assigned a value of 0–1 in Modde, wherein a value greater than 0.5 indicates good repeatability [14]. Modde also calculated the diagnostic parameters $R^{2}$ and *p*-value. The $R^{2}$, ranging from 0 to 1, indicates how close the calculated responses were to the experimentally measured responses [14]. The *p*-value was estimated by constructing the analysis of variance (ANOVA) table. A null hypothesis was set as follows Equation (3):

(3) $$H_{0}:\;\beta_{1}={\;\beta }_{2}=\beta_{3}={\;\beta }_{11}=\beta_{22}=\beta_{33}={\;\beta }_{12}={\;\beta }_{13}=\beta_{23}=0$$

If *p* was <0.05, the hypothesis was rejected. This condition would occur, when one of the coefficients was significantly different from zero and was therefore, significantly influencing the response y [18]. Thus, the factor, whose coefficient was significant, is a significant factor. The next procedure involved predicting the response window as a function of the factors. Therefore, response surfaces were plotted and analyzed as contour plots following the response surface methodology described in [14,18]. In this methodology, if the response surface was denoted by *η*, then it can be represented as follows Equation (4):

(4) $$\eta =E\left(y\right)=f\left(x_{1},x_{2},x_{3}\right)$$

Here, $f\left(x_{1},x_{2},x_{3}\right)$ represented the fitted regression model of Equation (2) (recall that fitting was performed by calculating the regression coefficients by the method of least squares). The plots were generated in Modde where $E\left(y\right)$ represented the expected response (the expected response was an expression for the mean response) of the regression model at the design space of the independent variables $x_{1},x_{2}$ and $x_{3}$. The plots were presented graphically where one x value was held constant and the remaining two x values were varied.

### 2.2. L-PBF

**Figure 1:**
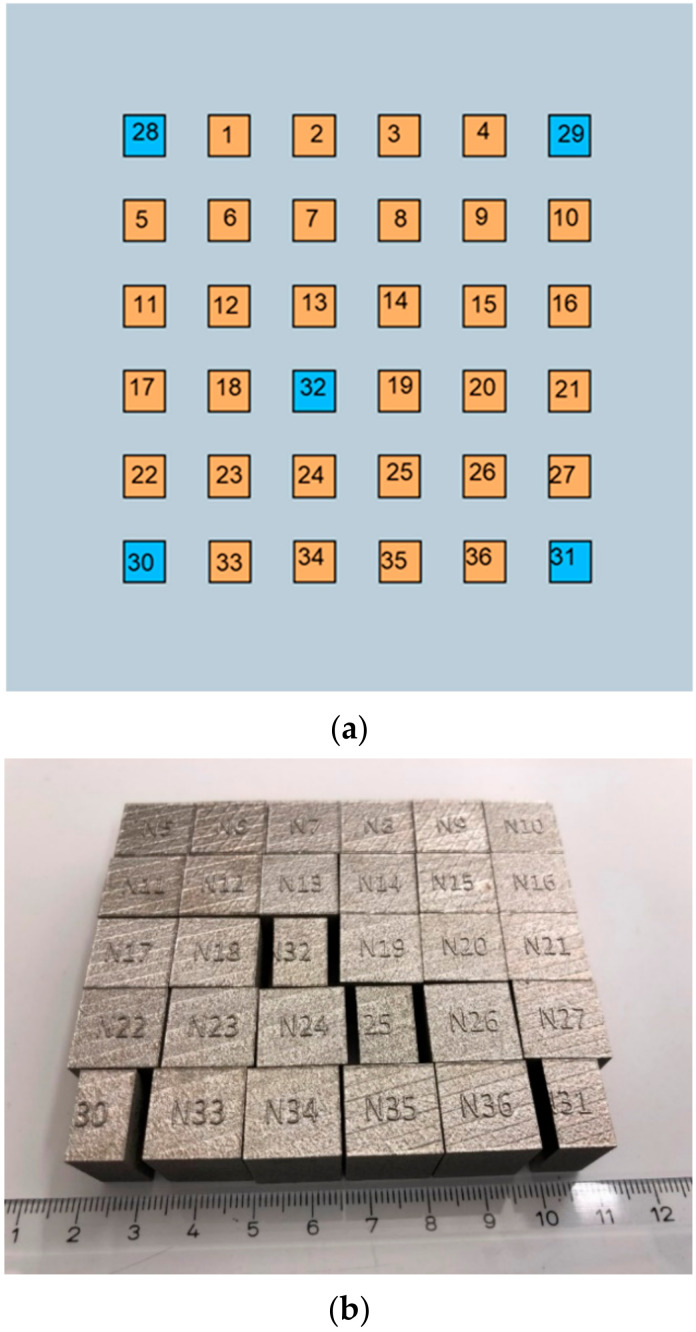
(**a**) Illustration of cube locations with the orange color representing experiments with different process parameter sets and blue color representing repeated experiments (replicates); (**b**) Some of the manufactured cubes.

Thirty cubes of dimensions 15 mm × 15 mm × 15 mm were manufactured with M290 equipment (Electro Optical Systems (EOS) GmbH, Krailling, Germany), according to the process parameters given in the first four columns of Table 1. Six more cubes (Samples 31–36) were manufactured, with hand-picked process parameters. These samples were later used to validate the results of the factorial design. The layer thickness was kept constant at 20 µm. Modde was used to produce the parameter matrix as explained in Section 2.1. The cubes were arranged as illustrated in Figure 1a. The blue colored cubes represented the replicated experiments (Samples 31 and 32 were also replicates), and the orange colored cubes were the experiments with varied process parameters. The replicates have a center point level, which meant that their values were at the midpoint of the levels of each factor. For example, the power level 195 J/s was the midpoint of 170 and 220 J/s. The arrangement of the replicates was made as illustrated, so that the response owing to the location of the replicates could be compared. The stripe scanning strategy was used, according to which when building a new layer, the scans were rotated 67° from the direction of the previously built layer. More details of the stripe pattern strategy can be found in [22]. There were no contour scans. Figure 1b shows some of the cube samples manufactured. Note that a cross section had been cut from, for example, Sample 31 for investigations, which is discussed in Section 2.3. The material used was gas atomized Alloy 247LC virgin powder, whose nominal composition is given in Table 2. The average particle size of the powder was approximately 30 µm and the powder had a certain porosity caused by argon gas entrapment and shrinkage. Details of the powder characterization are given in [23].

### 2.3. Void and Crack Quantification

Each of the cubes were cut and examined at approximately 5, 9, and 12 mm from an edge of the cube. Sample 31 in the bottom right corner of Figure 1b shows an example of the appearance of a cube after the first cross section was cut out. The investigated surface was parallel to the L-PBF build direction, which was from the bottom to the top of the cube. The cross sections were mounted in a hot mounting resin, ground, and polished in an automated grinding equipment. The samples were not etched. An optical microscope Zeiss Axio (Carl Zeiss Microscopy, GmbH, Göttingen, Germany) was used to capture nine images at 50× magnification; the images were spread over the entire area of each sample. The voids were quantified in the image analysis software Image J (Version 1.51, National Institutes of Health, Bethesda, MD, USA). A threshold was manually applied to exclude cracks from the image, so that only the voids were present. The total area of voids as a percentage of the area of the image was adopted a measure for the void content. The average void content of the images for all cross sections was gathered. In the crack density quantification, nine optical images, spread along the entire area of each sample were photographed, and the total crack length of all the cracks was measured. The measurements were made using the Zeiss application software of the microscope at 100× magnification. The crack density (1/mm) was calculated by dividing the total crack length by the total area of the micrographs to give the crack density.

### 2.4. Microhardness Measurement

The microhardness was measured in a Struers Duramin-40 tester (Struers Inc, Cleveland, OH, USA). A 0.5 kg test load was employed to make indentions at nine points in each sample with a dwell time of 10 s. The nine points were divided into three points each, distributed in the top, middle, and bottom of the samples. This was done to determine if the microhardness measurements varied along the height of each cube.

## 3. Results and Discussion

### 3.1. Voids

**Figure 2:**
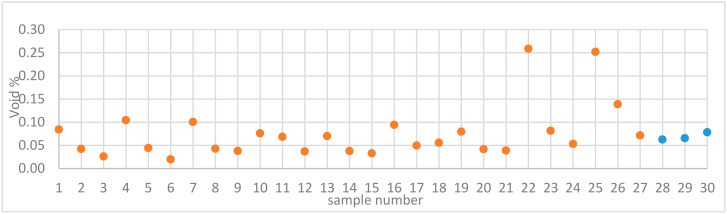
Void contents of samples manufactured by L-PBF (samples with varied parameters are in orange color, while replicates are in blue).

The void contents of Samples 1–30 are displayed in Figure 2. These data were taken directly from Table 1. The colors were chosen according to Figure 1a.

**Figure 3:**
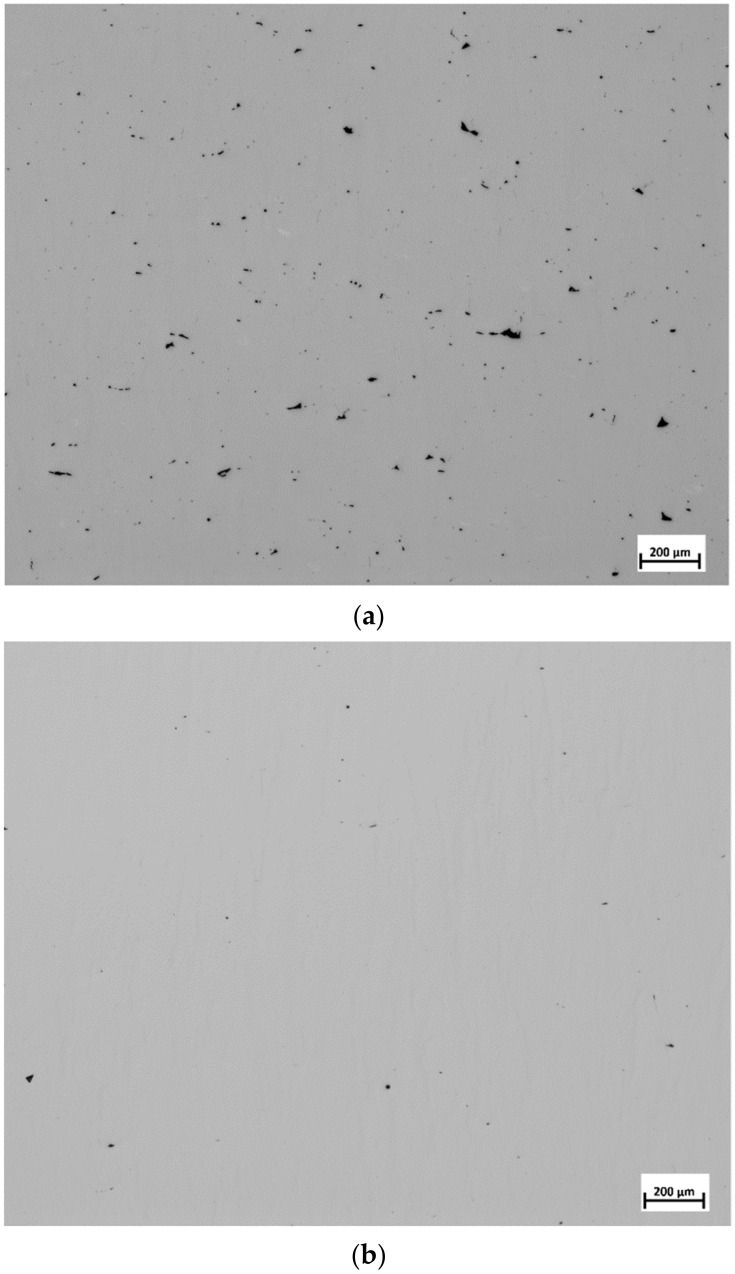
Typical optical micrographs showing noticeable difference in the void contents among the samples: (**a**) Sample 22; (**b**) Sample 28.

Sample 14 and its replicates displayed similar low void content values. This was reflected in the calculated repeatability value of 0.91. This indicates that the location of the cubes may not be influencing the void content result. This is vital to the study as it shows that the voids are linked to the process parameters alone and were independent of the location on the build plate. This need not be the case if the location-dependent thermal history influenced the void content as was observed in [24]. In an earlier study, the same EOS M290 equipment was used to manufacture samples of Alloy 247LC [25]. The energy density used in that study was 113.5 $J/{\mathrm{mm}}^{3}$, and the resultant void content was 0.04%, which was close to a void content of 0.06% displayed by Sample 18. It should be noted that sample 18 was manufactured with a similar energy density of 115 $J/{\mathrm{mm}}^{3}$. Several samples revealed low void content, i.e., less than 0.05% and hence, these samples could be considered as completely dense for many applications. Sample 22 displayed the highest void content, followed by Samples 25 and 26. Typical optical micrographs of Samples 22 and 28 (one of the replicates) are shown in Figure 3.

**Figure 4:**
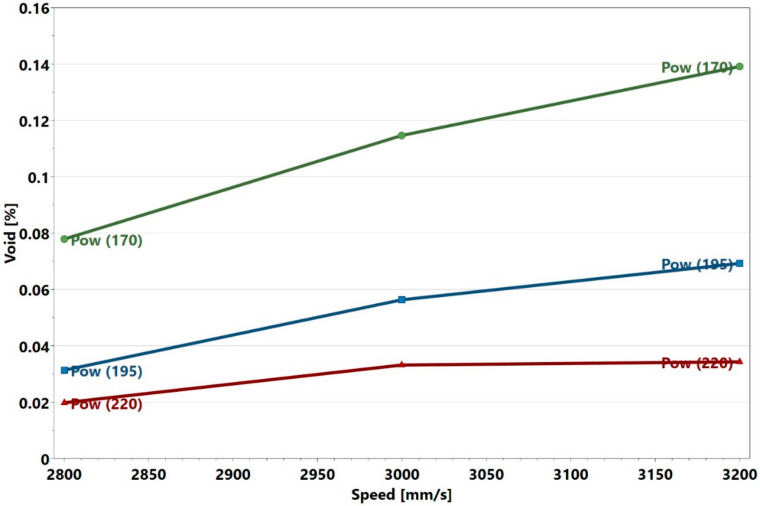
Power and speed interaction plot.

It is apparent that the void content was high in Sample 22 compared to that of Sample 28. The lack of fusion was observed to be the main cause for these voids. Lack of fusion is associated with a low energy density input. Under such a condition, full melting is not attained and only a small amount of liquid is formed, which inhibits wettability, thereby causing a lack of fusion [12]. Samples 22, 25, and 26 have energy densities of 71, 66, and 76 $J/{\mathrm{mm}}^{3}$, respectively. These values were the lowest among the energy densities calculated for all the samples. It was proposed that a critical energy density value existed where full densification would occur [2,12,26]. According to Carter, that value was 85 $J/{\mathrm{mm}}^{3}$ for nickel-based superalloy components manufactured by L-PBF [26]. In the present work, it was found that at and above 81 $J/{\mathrm{mm}}^{3}$, all the samples were at least 99.9% dense. This value was close to what was proposed by Carter. Below the energy density threshold, void content increased steeply. An energy density plot of the voids is presented at the end of Section 3.4. The interaction plot of power and speed on the void content was analyzed in Modde and is displayed in Figure 4.

**Figure 5:**
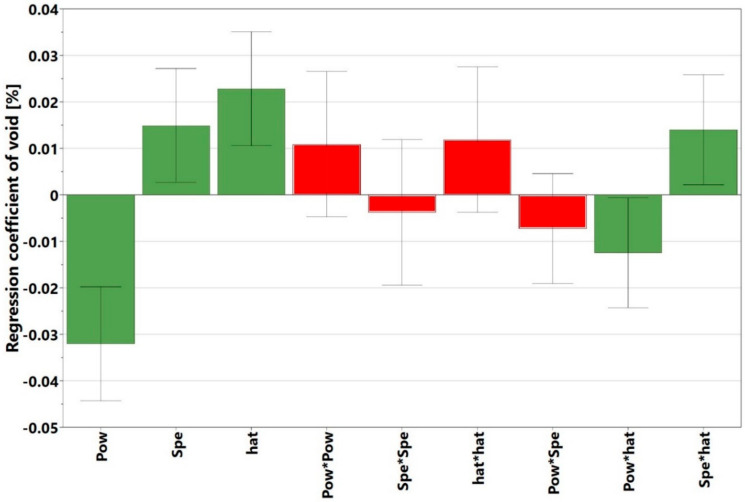
Regression coefficients for different factors and interaction of factors affecting void content (green bars indicate significant factors, while red bars indicate non-significant factors).

**Figure 6:**
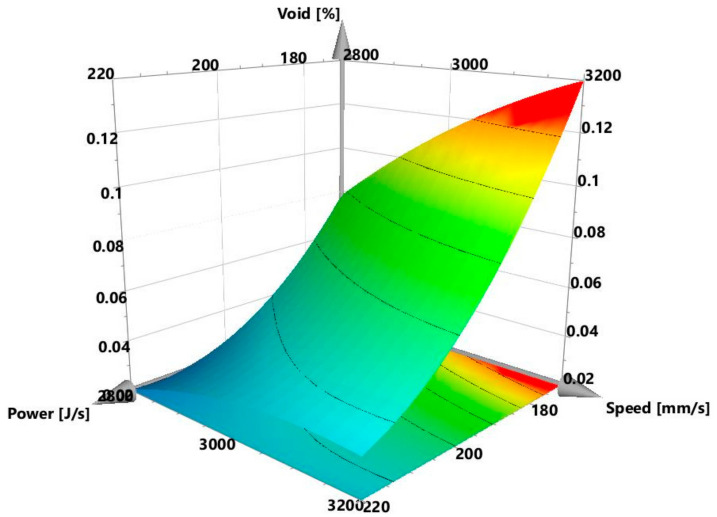
Response surface plot showing void content at different powers and speeds for a hatch of 30 µm.

The initial observation was that there was no noticeable interaction between the power and speed, as the red, blue, and green power plots were approximately parallel to one another. The explanation for this is presented later in this section and in Section 3.2, when the crack density interaction plots are discussed. Figure 4, however, shows that the void content increased with decreasing power and increasing speed. The *p*-value of the regression model of the present study was 0.000, which indicates that the model was statistically significant. It is important to know which of the process parameters had the strongest influence on the void formation, and whether there were any significant interactions among these parameters. To this end, the regression coefficients calculated in Modde, are presented in Table 3. The parameters that had statistically significant values (*p* < 0.05) are marked in green, while those that had non-significant values (*p* ≥ 0.05) are marked in red. All the coefficients are plotted in Figure 5 and shown together with their 95% confidence intervals. In line with the information presented in Table 3 and Figure 5, it can be interpreted that the regression coefficient for power was statistically highly significant and had the strongest influence on the void content. The interactions power*power, speed*speed, hatch*hatch, power*speed, were not statistically significant. This is evident from Figure 5, wherein the non-significant parameters marked in red had zero included in their confidence intervals. The parameters in green were significant and influenced the void content. This interpretation was consistent with the null hypothesis given in Section 2.1. An example of a response surface plotted for void content is displayed in Figure 6. Here, the void content is observed to be varying with power and speed. The hatch was constant at 30 µm. The lines of constant voids were projected on the power and speed plane as contour plots. Similar surface response and corresponding contours were plotted for hatch distances of 20 and 40 µm but were not shown here for brevity. Contour plot examples were displayed in Section 3.4 where the influence of power, speed and hatch on the void can be simultaneously observed.

**Figure 7:**
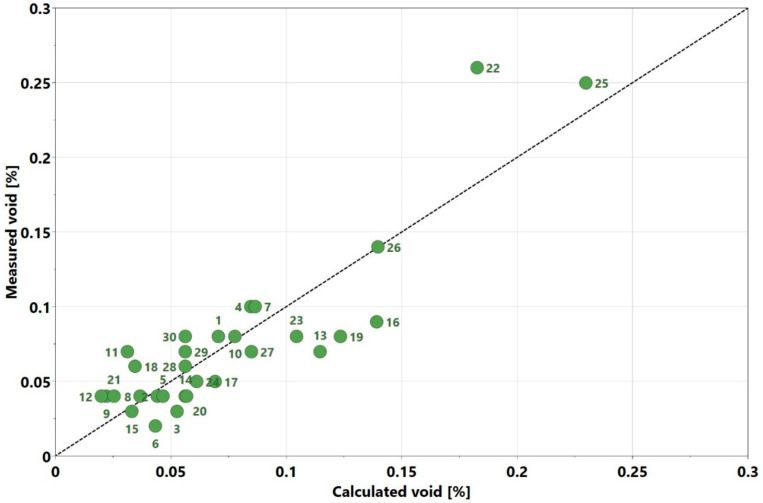
Comparison of experimentally measured void content with calculated void content from the regression model.

The next step is to know how the experimentally measured void content compared with the calculated results of the regression model. The regression model was the following. $y=\;0.0563162-\;0.0320426x_{1}+\;0.014924x_{2}+\;0.0228249x_{3}+\;0.0108931x_{1}^{2}-\;0.00378128x_{2}^{2}\;+$ $0.0118714x_{3}^{2}-0.00726209x_{1}x_{2}-0.0124493x_{1}x_{3}+0.0140055x_{2}x_{3}$. Recall that the coefficients had confidence intervals and the x variables were defined on a coded scale as explained in Section 2.1. The $R^{2}$ value was 0.78, which indicates that the experimental results were reasonably close to the calculated values. A plot comparing the measured and calculated void contents is displayed in Figure 7. It appears that the fit was better when the values were <0.1%; however, the overall fit was still significantly close to the 1:1 line (the line crossing the points where the calculated values are equal to the measured values).

### 3.2. Cracks

**Figure 8:**
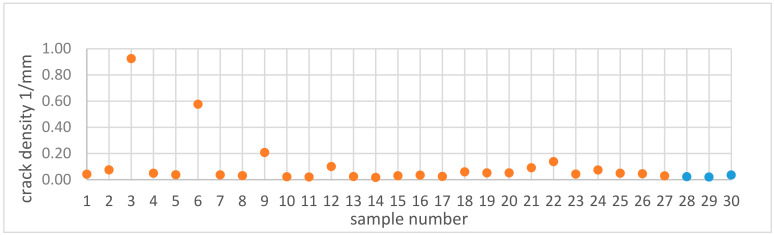
Crack density of samples manufactured by L-PBF (samples with varied parameters are in orange color, while replicates are in blue).

**Figure 9:**
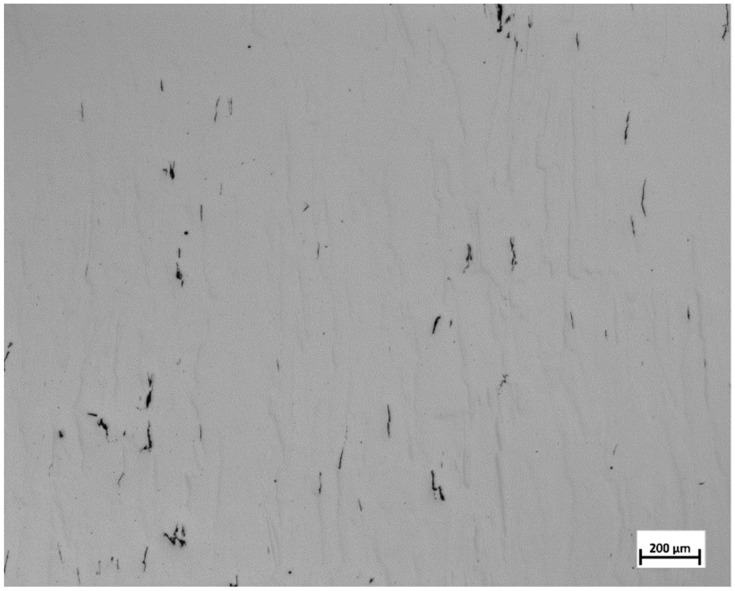
Typical micrograph (of Sample 3) showing prevalence of cracks.

**Figure 10:**
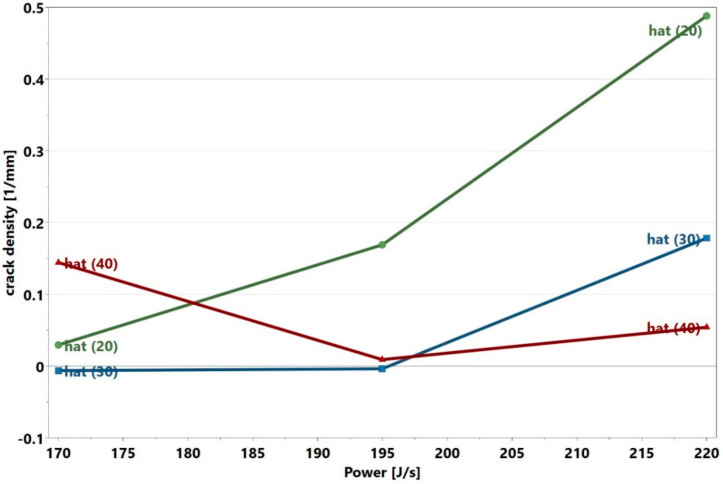
Power and hatch interaction plot.

The crack density, as seen in Table 1 and displayed in Figure 8, was lower compared to that reported by Carter [2]. Sample 14 and its replicates displayed crack density values which were close to one another, which again indicated that the location may not influence the crack density. This was evident with the repeatability value of 0.99. Unlike in the study by Carter et al., many samples in the present study had crack densities close to zero, as shown in Figure 8. Samples 3 and 6 had the highest crack densities, which corresponded to the highest energy density values. Figure 9 shows a typical optical micrograph (taken of Sample 3) displaying the cracks. This micrograph could be compared to that of Sample 28, which had a crack density close to zero, as depicted in Figure 3. It was observed that samples 3 and 6 with high crack density displayed an energy density trend opposite to that displayed by samples 22, 25 and 26 with high void content. It appeared that high energy density promoted cracking—an observation that agreed with those from the literature [2,27]. This trend was observed above 163 $J/{\mathrm{mm}}^{3}$ where a steep increase in crack density occurred (see the end of Section 3.4). The interaction analysis in Modde for power and hatch can be used to illustrate this trend, as shown in Figure 10.

**Figure 11:**
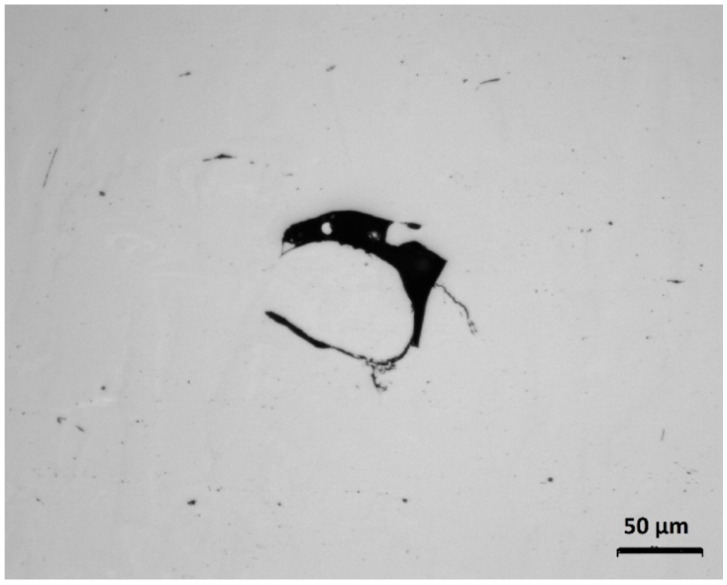
Micrograph of Sample 22 showing a crack (indicated by the arrow) initiated at the edge of a lack of fusion void.

What is immediately evident is the significance of power*hatch interaction, which is the reason for the plots crossing each other at some points. It shows that the crack density trend (the slope of each plot) of the power depends on the level of the hatch. The void content plots of the power and speed shown earlier in Figure 4 did not cross one another because their interactions were not significant. It is seen in Figure 10 that the crack density reduced with reduced power at power levels between 195 and 220 J/s. The rate of reduction, however, was less at power levels < 195 J/s, as displayed in the 20 µm hatch case. The crack density was almost constant at 30 µm hatch and eventually increased at 40 µm hatch. This characteristic of the graph at low power and large hatch was because the cracks started to appear when the lack of fusion became higher. The sharp corners of the lack of fusion voids induced high stress concentrations, which promoted cracking [28]. This is displayed in Sample 22, depicted in Figure 11.

**Figure 12:**
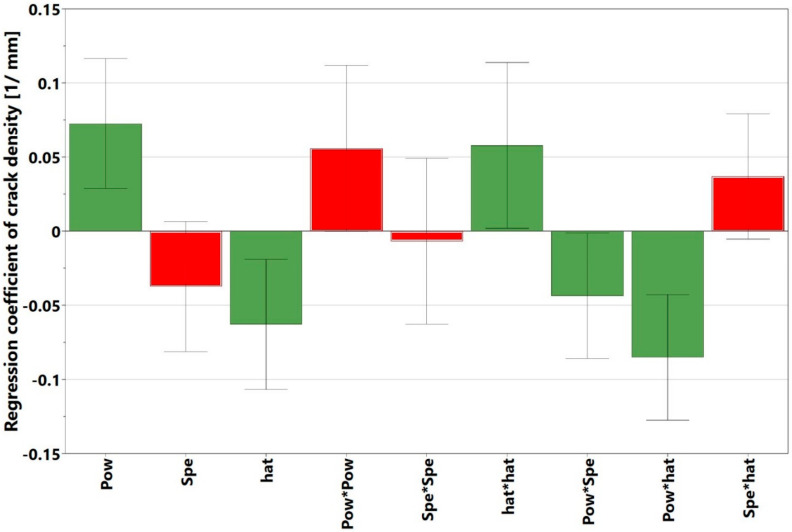
Regression coefficients for different factors and interaction of factors affecting the crack density (green bars indicate significant factors, while red bars indicate non-significant factors).

**Figure 13:**
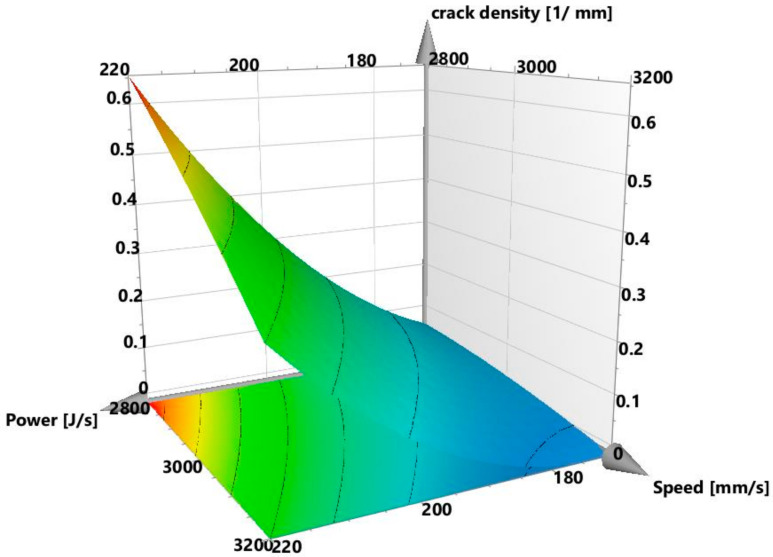
Response surface plot showing crack density at different powers and speeds for a hatch of 20 µm.

**Figure 14:**
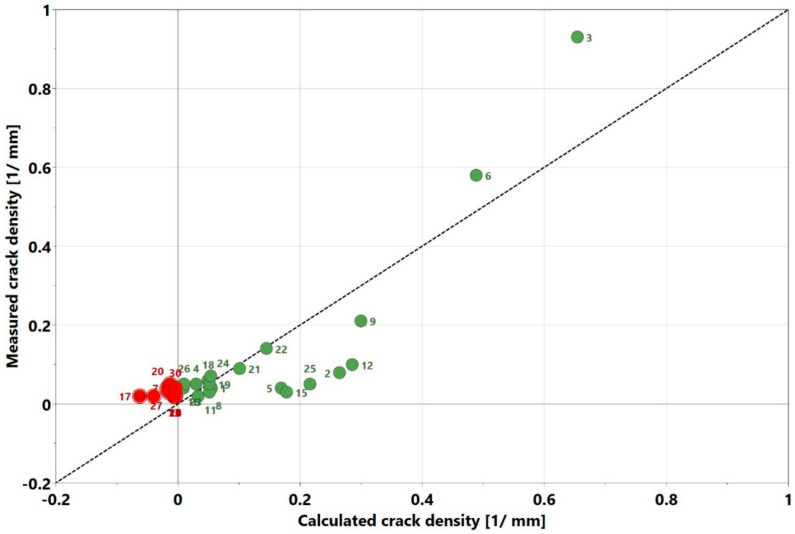
Comparison of experimentally measured crack density with calculated values from regression model (red data points indicate calculated point with negative values of crack density).

The origins of some of the cracks, however, were not so evident and require further investigations. The regression coefficient chart is displayed in Figure 12. Here, the interaction parameter power*hatch was the strongest and most statistically significant process parameter influencing the crack density. Other significant and non-significant factors could be identified using the approach described in the section on void content. The *p*-value was 0.000 and the response surface plot is also shown in Figure 13, which displays the variation of crack density with power and speed at a constant hatch of 20 µm. The general trend of a high cracking density with high power and low speed is clear and is also visible in the contour plots for crack density in Section 3.4. The plot of the experimentally measured crack density values, when compared to the calculated results is displayed in Figure 14. The regression model was fitted as $y=\;-0.00394012+\;0.07258x_{1}-\;0.0373897x_{2}-\;0.0629027x_{3}+\;0.055843x_{1}^{2}-0.00680932x_{2}^{2}\;+$ $0.0578009x_{3}^{2}-0.0436657x_{1}x_{2}-0.0852521x_{1}x_{3}+0.0369079x_{2}x_{3}$. Even though the $R^{2}$ of the model was reasonably high at 0.75, it can be noticed that the calculated results did not accurately match the measured results. The model exhibited lack of fit; this was indicated in the ANOVA table. It is noticeable in Figure 14 that negative values were predicted for the crack density. Its physical interpretation is not that negative crack content existed. In fact, the measured values were close to zero, which along with a lack of fit, made it numerically possible for negative values to be generated.

### 3.3. Microhardness

**Figure 15:**
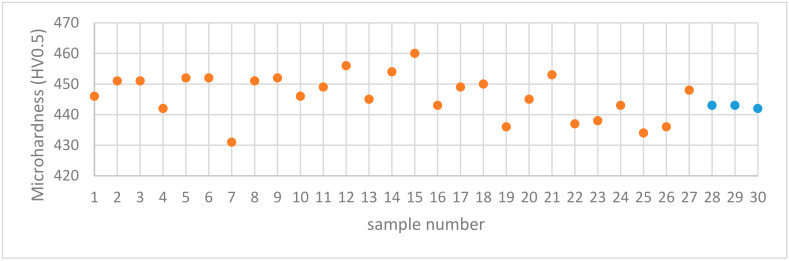
Microhardness of samples manufactured by L-PBF (samples with varied parameters are in orange, while replicates in blue).

The microhardness of all the samples was measured and the results are presented in Figure 15. The microhardness values ranged between 431 and 460 HV0.5.

**Figure 16:**
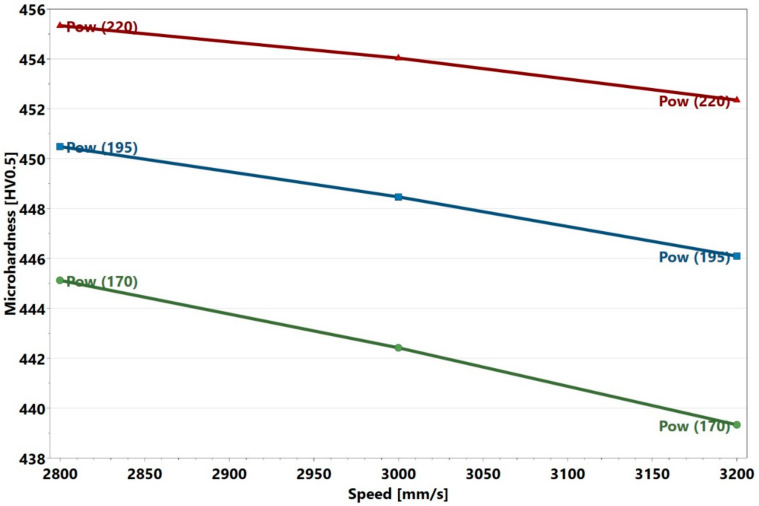
Power and speed interaction plot.

**Figure 17:**
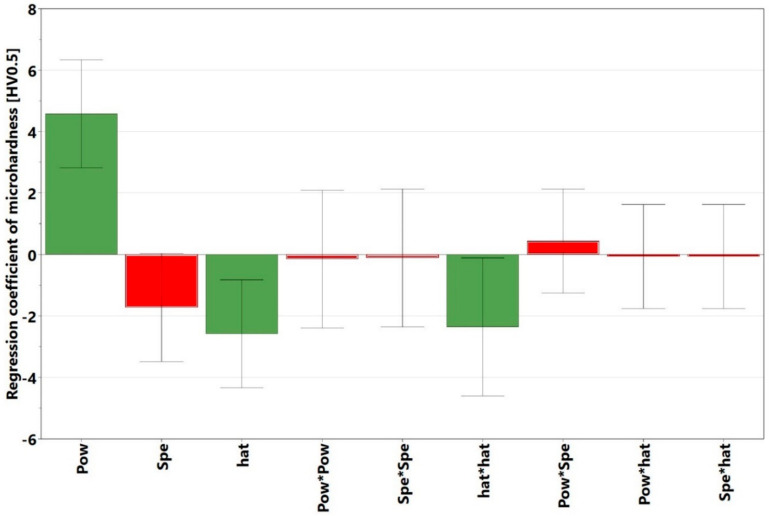
Regression coefficients of different factors and interaction of factors affecting the microhardness (the green bars indicate significant factors, while the red ones indicate non-significant factors).

**Figure 18:**
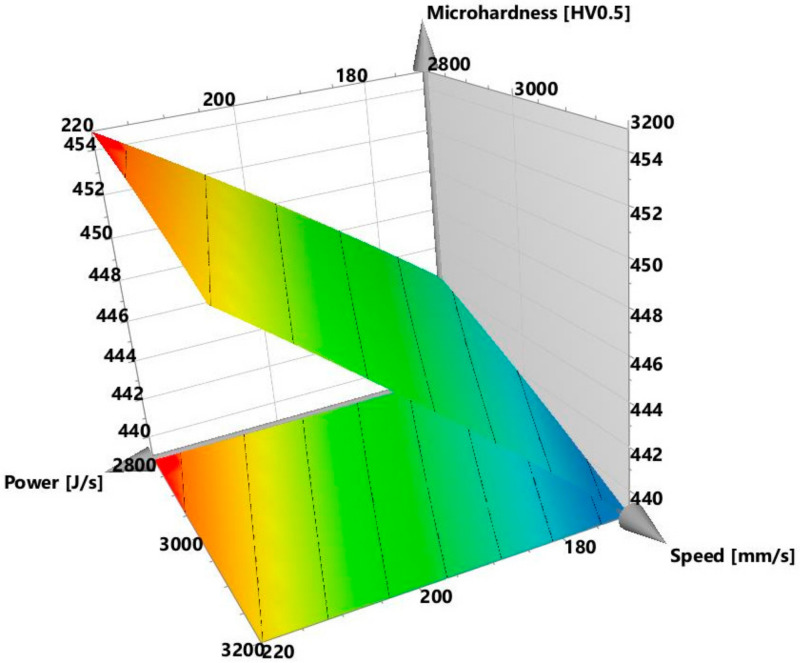
Response surface plot showing microhardness at different powers and speeds for a hatch of 20 µm.

**Figure 19:**
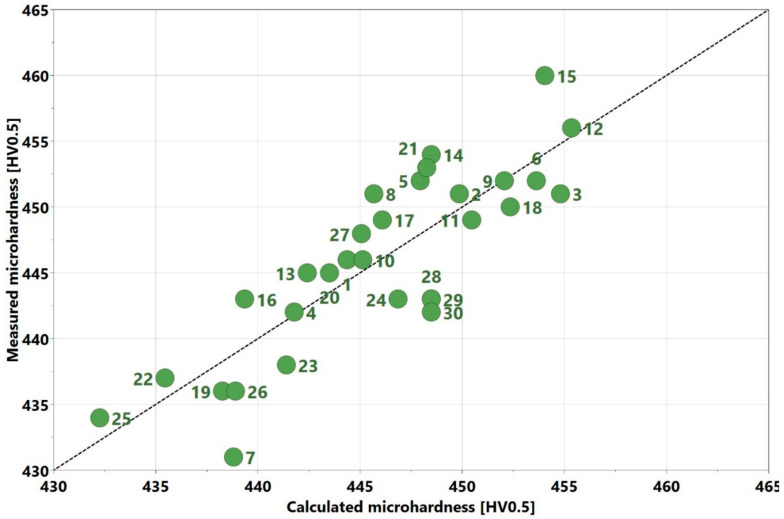
Comparison of experimentally measured microhardness to the calculated values from the regression model.

The reported values of microhardness of cast Alloy 247 samples were within the range of 430–456 HV0.5 [29]. The present L-PBF manufactured Alloy 247LC samples, thus demonstrated microhardness close to that of the cast samples, which indicates that L-PBF compares favorably with the conventional methods of manufacturing. The highest microhardness of the present samples was also close to 463 HV0.5 reported for the L-PBF of alloy 247 [19]. Alloy 247 has a higher carbon content than Alloy 247LC. The average microhardness measurement in a previous L-PBF Alloy 247LC study mentioned in Section 3.1 [25] was 421 HV0.5, which was lower than the 450 HV0.5 obtained from a similar energy density sample (Sample 18). The replicates, i.e., Samples 28, 29, and 30 had similar values, but they differed from Sample 14. The repeatability value, which was 0.34, was thus low. The other replicates, Samples 31 and 32 outside the factorial design had similar values as did Samples 28, 29, and 30 (refer to Table 4 and Table 5 in Section 3.4). Thus, this suggests that the samples may actually be repeatable. The reason why the replicates differed from Sample 14 could not be ascertained and would need a more detailed microstructural investigation. The microhardness plot portrayed that the values were close to one another for all the samples. As can be seen, the difference between the highest and lowest microhardness values was 29 HV0.5. Despite this, the analysis in Modde still portrayed that a relationship existed, which was supported by a strong statistically significant *p*-value of 0.001. Figure 16 displays the interaction plot of power and speed. Here, it is evident that high power and low speed led to higher microhardness. There was no significant power and speed interaction, which was also evident in Figure 17. The predictions of the response surface plot are displayed in Figure 18, which also corresponds to the observations made from Figure 16. Power was the most statistically significant process parameter, as displayed in Figure 17. It appeared there was a trend of low microhardness as void content decreased. It was noticeable that the samples with the highest void content and lowest energy density, as discussed earlier (Samples 22, 25, and 26) demonstrated relatively low microhardness. The samples with the highest crack density (Samples 3 and 6) demonstrated microhardness values that belonged to the top 33% of all the microhardness values. A distinct relationship between microhardness and crack density, however, was not evident. It is known that the primary strengthening mechanism in conventionally processed nickel-based superalloys—for example, Alloy 247LC—is by the precipitation of the ordered γ’ [30,31]. Findings from literature also confirm the presence of γ’ in the as-built condition of L-PBF-manufactured Alloy 247LC components [19,20,21]. This aforementioned mechanism may depend on the process parameters and will affect the microhardness of the samples (different process parameters result in different thermal treatments, which may result in different sizes and amounts of γ’ precipitation). Correspondingly, the cracking behavior may be influenced. For example, a top layer may heat the preceding layer, which may cause a stress relief to happen in the temperature range, in which γ’ is precipitated, thereby causing a strain age cracking. The presence of a high volume fraction of γ’ may also make it susceptible to constitutional liquation, causing hot cracks, as suggested by [32]. The microstructure characteristics and crack mechanism would warrant a detailed microscopic evaluation and will be part of a future study. It was also of interest to know if the microhardness changed along the height of the sample, because of the layer-by-layer melting approach. Therefore, the microhardness values obtained in the top, middle, and bottom of each sample were compared. The results showed that these values were close. Wang et al. [21] showed that the γ’ sizes were similar in the top layer and the bulk of the L-PBF-manufactured Alloy 247LC, which implies that the microhardness may be similar. This may not be the case if the local thermal history in the sample leads to different γ’ sizes or if γ’ coarsening occurs, as was observed in [33,34]. A comparison of the measured microhardness to the calculated values is displayed in Figure 19. The regression model was fitted as $y=\;448.474+\;4.5794x_{1}-\;1.72675x_{2}-2.58229x_{3}-0.153924x_{1}^{2}-0.116772x_{2}^{2}-\;2.35728x_{3}^{2}\;+$ $0.434383x_{1}x_{2}-0.0677355x_{1}x_{3}-0.0696204x_{2}x_{3}.$ Here, the $R^{2}$ value was 0.71; the points are relatively close and uniformly dispersed around the 1:1 line. This demonstrates a relatively good fit.

### 3.4. Prediction of Process Parameter Window

**Figure 20:**
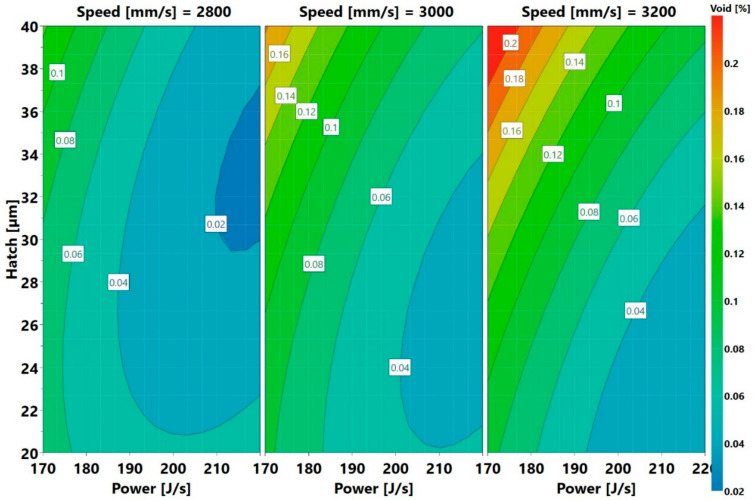
Void contour plots providing predictions of where low void fraction is likely to be present.

To predict the windows of power, speed, and hatch, where low void content and crack density can be found, three perspectives were defined. In the first two, it was of interest to determine the process parameters, for which either a low void content or low crack density exists. The third perspective was to determine the process parameters, corresponding to which a combination of low void content and low crack density exists. The first approach, low void content, can be analyzed as follows, using a 4D void contour plot displayed in Figure 20.

**Figure 21:**
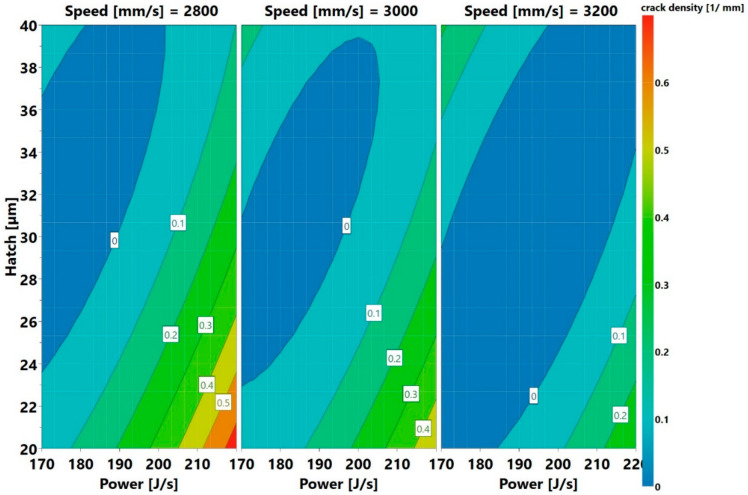
Crack density contour plots providing predictions of where low crack density is likely to be present.

The plot shows the variation of hatch distance with power at three different speeds. The lines of constant voids are marked on the plot. The operating window of low void content, for example areas with a void content of less than <0.04 (colored light blue and dark blue), are observed at the right-hand side of every speed plot. The area moves from the bottom right at a speed of 3200 mm/s upwards as the speed reduces. Thus, the lowest voids were found at the high power lines of 2800 mm/s speed. Notice that the low void content region extended almost throughout the length of the hatch axes. The plot also shows that low void content areas could be found outside the present parameter windows. This region should be within the blue colored region, away from the right side of the contour maps. An example of a prediction of a new process parameter set that is likely to produce a low void content is 240 J/s, 2800 mm/s, and 30 µm. Reducing the speed to 2600 mm/s and operating at the present power and hatch values are also likely to increase the blue area. It should, however, be remembered that speed had a lesser influence than power in the void content, as displayed in Figure 5. The prediction using the low void content approach implies that there would be cracks remaining. These cracks, anyway, would have to be removed by some viable methods. As the high stresses prevalent in the L-PBF process are one of the main causes of cracking, methods that reduce the stress could eliminate the cracks. This can be achieved, for example, by preheating the substrate, as proposed in [35]. Hot isostatic pressing (HIP) has also been demonstrated to close the cracks as seen from microstructure evaluations [13,25,36]. However, the creep life of HIPed L-PBF samples is very low, when compared to cast parts, even after undergoing solutioning and ageing heat treatments [2]. This suggests that the cracks may not be fully closed or that the regions of the cracks may have low ductility. The eventual solution to this problem may involve compositional modification. An example of such modification in Inconel 738LC was proposed in [37]. The second approach of lowering the crack density is considering the 4D contour plots for crack density, as displayed in Figure 21.

**Figure 22:**
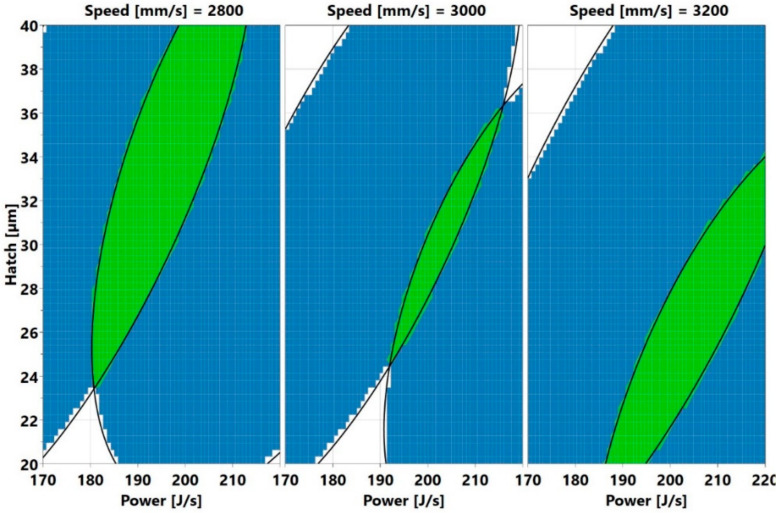
Hatch versus power and speed plots (the green areas represent process parameter sets. which yield low void content and low crack density, i.e., less than 0.05% and 0.05/mm of void fraction and crack density, respectively).

Here the low crack density area (shaded in blue) is shifting from the top left to the middle as the speed increases. The blue region is wider at 3200 mm/s. Even here, low crack density process parameter sets, outside the present window exist. An example is the parameter set 145 J/s, 3200 mm/s, and 20 µm. The third approach requires plotting a sweet spot where both low porosity and low crack density could be found. The criterion used for this was that both porosity and crack density should be less than 0.05% and 0.05/mm, respectively. The sweet spots are the green areas displayed in Figure 22.

Here, blue means low void content or crack density, while white means neither low void content nor low crack density.

New parameter sets for void content and crack density could be investigated by designing new experiments that are based on the present predictions. This will be carried out in a future study. Currently, the six samples, whose parameters were chosen manually, and which were not included in the factorial design, could be used to validate the present model. Their results are displayed in Table 4 and Table 5. Table 4 shows the process parameters, while Table 5 shows the measured values (in line with Section 2.3 and Section 2.4), and the model-calculated values (in line with Section 2.1). The predicted microhardness was close to the measured values. However, the predicted void content and crack density showed some disparity when compared to the measured values. Nevertheless, the measured and calculated values were close from an application perspective. For example, Sample 34 displayed a possibly large disparity in the void content (50% higher predicted value than the measured value). This difference was, however, not significant when sample densities (a measured density of 99.94% to predicted density of 99.91%) were compared. The negative values in the predicted crack density of Samples 33 and 34 reflected the lack of fit in the model. This is not a physical reality, as explained in the discussion of Figure 14. Such a situation was also observed in [2], [15]. It was discussed earlier that one of the main challenges during the L-PBF processing of Alloy 247LC was cracking. Even though crack density close to zero could be obtained in the experiments, small cracks could still be found in reality. These cracks have a high potential of propagating and deteriorating the mechanical properties. The remaining voids may not pose serious problems and could be successfully removed by HIP. It may be recalled that a number of samples had very low void content (less than 0.05% porosity), which is considered dense for its intended applications in gas turbine blades.

**Figure 23:**
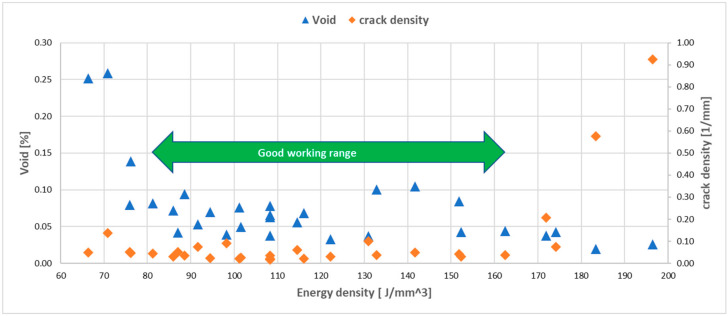
Energy density working range, in which low void content and crack density are found.

It is also useful to examine the energy density range, in which both low void content and crack density could be found. As the analysis was not included in the DOE factorial design in Modde, a map was created in excel to show the trend. It may be recalled that the layer thickness was included in the energy density. A good working range becomes evident when the measured void content and crack density values are plotted over energy density, as displayed in Figure 23. The arrow identifies an energy density range between 81 and 163 $J/{\mathrm{mm}}^{3}$, where both void content and crack density were relatively low. Outside this range, either the void content (on the left side of the arrow) or the crack density (on the right side of the arrow) began to rise steeply.

## 4. Conclusions

The main conclusions from this study can be summarized as follows:

- Highly dense Alloy 247LC samples with low crack density were obtained with appropriately selected L-PBF process parameters.
- High energy density (above 163 $J/{\mathrm{mm}}^{3}$), defined by power, speed and hatch, led to a steep increase in crack density. Low energy density (below 81 $J/{\mathrm{mm}}^{3}$) led to a steep increase in void content.
- There was no strong trend for the relation between the microhardness and crack density; however, relatively high microhardness was obtained in the two samples that demonstrated the highest crack density. A trend of low microhardness was displayed in samples with high void content.
- Power was the strongest process parameter affecting the void content, while the interaction of power and hatch was the strongest process parameter influencing the crack density. Finally, power was the strongest process parameter influencing the microhardness.
- Process parameter windows were obtained for the lowest crack density and/or lowest void content. New process parameters for low void content and crack density were predicted.

A proposal for future work is given in the following.

- The predicted process windows given in Section 3.4 indicted that other sets of parameters that may produce low voids and cracks exist. Therefore, the presented process windows can be used as a guide to expand the DOE for further analysis.
- New responses connected to the microstructure and crack formation mechanism in the samples can be measured and statistically analyzed across the 30 samples (or a new DOE samples) using the same approach in the present paper. The following responses are proposed.
  ○ Grain size;
  ○ Size and volume fraction of phases and precipitates;
  ○ Extent of element segregation;
  ○ Creep life and strain.
- A well-designed scanning strategy mitigates the stress and may reduce the cracks. The role of different scanning strategies on the cracks should be investigated.
- It is important to detect the cracking mechanism(s) and propose solutions. Thus, the microstructure should be investigated using advance microscopy—for example, high resolution scanning electron microscopy.

## Figures and Tables

**Table 1:** Full factorial design and measured responses.

Experiment No	Power (J/s)	Speed (mm/s)	Hatch (µm)	Energy Density $\left(J/mm^{3}\right)\;*$	Void (%)	Crack Density (1/mm)	Microhardness (HV0.5)
1	170	2800	20	152	0.08	0.04	446
2	195	2800	20	174	0.04	0.08	451
3	220	2800	20	196	0.03	0.93	451
4	170	3000	20	142	0.10	0.05	442
5	195	3000	20	163	0.04	0.04	452
6	220	3000	20	183	0.02	0.58	452
7	170	3200	20	133	0.10	0.04	431
8	195	3200	20	152	0.04	0.03	451
9	220	3200	20	172	0.04	0.21	452
10	170	2800	30	101	0.08	0.02	446
11	195	2800	30	116	0.07	0.02	449
12	220	2800	30	131	0.04	0.10	456
13	170	3000	30	94	0.07	0.02	445
14	195	3000	30	108	0.04	0.02	454
15	220	3000	30	122	0.03	0.03	460
16	170	3200	30	89	0.09	0.04	443
17	195	3200	30	102	0.05	0.02	449
18	220	3200	30	115	0.06	0.06	450
19	170	2800	40	76	0.08	0.05	436
20	195	2800	40	87	0.04	0.05	445
21	220	2800	40	98	0.04	0.09	453
22	170	3000	40	71	0.26	0.14	437
23	195	3000	40	81	0.08	0.04	438
24	220	3000	40	92	0.05	0.07	443
25	170	3200	40	66	0.25	0.05	434
26	195	3200	40	76	0.14	0.05	436
27	220	3200	40	86	0.07	0.03	448
28	195	3000	30	108	0.06	0.02	443
29	195	3000	30	108	0.07	0.02	443
30	195	3000	30	108	0.08	0.04	442

* This is the energy density which includes a layer thickness of 20 µm.

**Table 2:** Nominal composition of Alloy 247LC (wt.%).

C	Cr	Ni	Co	Mo	W	Ta	Ti	Al	B	Zr	Hf
0.07	8.1	Bal	9.2	0.5	9.5	3.2	0.7	5.6	0.015	0.015	1.4

**Table 3:** Coefficients and *p*-values of regression constants.

Factor	Constant	Coefficient	*p*-Value
	$\beta_{0}$	0.0563162	0.000151063
Power	$\beta_{1}$	−0.0320426	0.0000245546
Speed	$\beta_{2}$	0.014924	0.0195258
Hatch	$\beta_{3}$	0.0228249	0.000923855
power*power	$\beta_{11}$	0.0108931	0.162019
speed*speed	$\beta_{22}$	−0.00378128	0.619757
hatch*hatch	$\beta_{33}$	0.0118714	0.129252
power*speed	$\beta_{12}$	−0.00726209	0.215015
power*hatch	$\beta_{13}$	−0.0124493	0.0401212
speed*hatch	$\beta_{23}$	0.0140055	0.022656

**Table 4:** Process parameters of the additional six samples.

Experiment	Power (J/s)	Speed (mm/s)	Hatch (µm)	Energy Density $\left(J/mm^{3}\right)\;*$
31	195	3000	30	108
32	195	3000	30	108
33	180	2800	30	107
34	180	3000	30	100
35	210	2800	30	125
36	210	3000	30	117

* This is the volumetric energy density, which includes a layer thickness of 20 µm.

**Table 5:** Measured and calculated values of the additional six samples.

Experiment	Measured Voids [%]	Predicted Voids [%]	Measured Crack Density (1/mm)	Predicted Crack Density (1/mm)	Measured Microhardness (HV0.5)	Predicted Microhardness (HV0.5)
31	0.09	0.06	0.02	0	444	448
32	0.05	0.06	0.02	0	445	448
33	0.07	0.05	0.01	−0.03	443	447
34	0.06	0.09	0.02	−0.02	444	445
35	0.02	0.02	0.03	0.16	449	453
36	0.03	0.04	0.02	0.08	450	452
